# The *Clostridium difficile* Protease Cwp84 Modulates both Biofilm Formation and Cell-Surface Properties

**DOI:** 10.1371/journal.pone.0124971

**Published:** 2015-04-29

**Authors:** Véronique Pantaléon, Anna Philibertine Soavelomandroso, Sylvie Bouttier, Romain Briandet, Bryan Roxas, Michele Chu, Anne Collignon, Claire Janoir, Gayatri Vedantam, Thomas Candela

**Affiliations:** 1 EA4043, Faculté de Pharmacie, Université Paris Sud, Châtenay-Malabry, France; 2 INRA, UMR1319, Micalis, Jouy-en-Josas, France; 3 AgroParisTech, UMR Micalis, Massy, France; 4 School of Animal and Comparative Biomedical Sciences, Department of Immunobiology, University of Arizona, Tucson, Arizona, the United States of America; 5 Southern Arizona VA Healthcare System, Tucson, Arizona, the United States of America; Universidad de Costa Rica, COSTA RICA

## Abstract

*Clostridium difficile* is responsible for 15-20% of antibiotic-associated diarrheas, and nearly all cases of pseudomembranous colitis. Among the cell wall proteins involved in the colonization process, Cwp84 is a protease that cleaves the S-layer protein SlpA into two subunits. A *cwp84* mutant was previously shown to be affected for in vitro growth but not in its virulence in a hamster model. In this study, the *cwp84* mutant elaborated biofilms with increased biomass compared with the parental strain, allowing the mutant to grow more robustly in the biofilm state. Proteomic analyses of the 630*Δerm* bacteria growing within the biofilm revealed the distribution of abundant proteins either in cell surface, matrix or supernatant fractions. Of note, the toxin TcdA was found in the biofilm matrix. Although the overall proteome differences between the *cwp84* mutant and the parental strains were modest, there was still a significant impact on bacterial surface properties such as altered hydrophobicity. In vitro and in vivo competition assays revealed that the mutant was significantly impaired for growth only in the planktonic state, but not in biofilms or in vivo. Taken together, our results suggest that the phenotypes in the *cwp84* mutant come from either the accumulation of uncleaved SlpA, or the ability of Cwp84 to cleave as yet undetermined proteins.

## Introduction

Biofilms are organized microbial communities, usually formed on abiotic or natural surfaces, where organisms are embedded into a matrix composed of extracellular polymers. These polymers may be composed of polysaccharides, DNA or proteins. For bacteria, the biofilm lifestyle is now increasingly appreciated as being predominant [1], since it enables protection from environmental insults, and may thus facilitate pathogenic mechanisms [2].

In anaerobic bacteria, biofilm formation has not been extensively studied, but is likely crucial to the long-term persistence of clinically-relevant pathogens such as *Clostridium difficile*. This bacterium is a spore-forming, obligate anaerobe, responsible for 15–20% of antibiotic-associated diarrheas, and nearly all cases of pseudomembranous colitis [3]. *C*. *difficile* infection (CDI) rates (primary and recurrent) have increased worldwide since the early 2000s, and have had major impact on healthcare costs [4]. This increase of CDI rates was associated with the emergence of epidemic strains such as the BI/NAP1/027 strains [5]. However, the basic pathogenic mechanisms of these *C*. *difficile* strains has not been elucidated, and it is now clear that CDI severity cannot be solely attributed to increased levels of the major intoxicants TcdA and TcdB.

*C*. *difficile* expresses proteins involved in mediating bacterial attachment to host cells, and thus facilitating colonization. Several of these molecules belong to the so-called Cell Wall Protein (Cwp) family [6]. Among them, SlpA is an abundant factor, expressed as a precursor protein that is cleaved into high molecular-weight (HMW-SLP, also called P47) and low molecular-weight (LMW-SLP, also called P36) subunits, which ultimately assemble in paracrystalline architecture (S-layer) on the bacterial surface. The protease responsible for SlpA processing is Cwp84, which also possesses the ability to cleave the eukaryotic extracellular matrix proteins fibronectin, laminin and vitronectin [7,8]. In the bacterium, Cwp84 is processed, exported to the surface, and its N-terminal signal peptide removed. Associated to the S-layer, this form is able to cleave SlpA. A *cwp84* mutant was recently constructed [9,10] and exhibited growth and colony morphology defects, but was not affected in virulence in a hamster model [10].

To date, the contribution of biofilms to CDI development or persistence has been unclear. *C*. *difficile* can form biofilms in vitro ([11–14], for review see [15]), but the robustness varies between clinical isolates. In the outbreak-associated R20291 strain, among the tested mutants *spo0A*, *luxS* and *cwp84* exhibited a defect in biofilm formation [11,12]. LuxS is involved in quorum sensing, and indirectly drives biofilm formation of most of bacteria, and the observed phenotype was therefore expected [16]. In contrast, the role of the Cwp84 protein in biofilm formation remained unclear. The aim of this study was to evaluate the contribution of *cwp84* to *C*. *difficile* biofilm formation in the type strain 630Δ*erm*.

## Results

### Disruption of *cwp84* promotes biofilm formation

**Fig 1:**
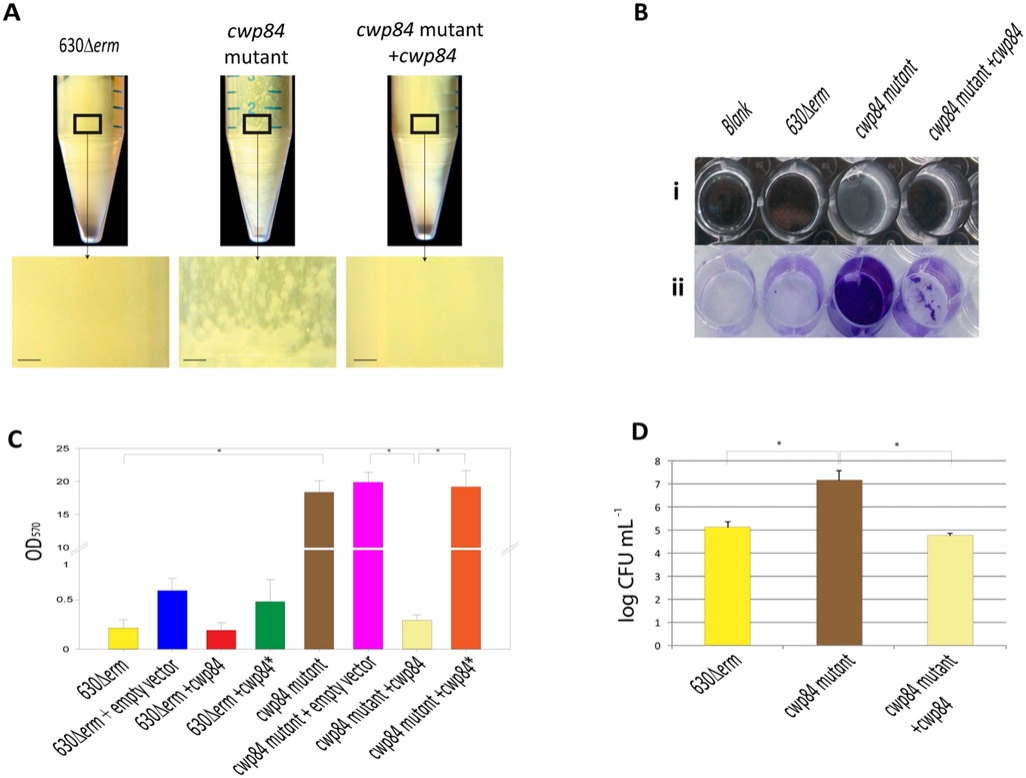
The *cwp84* mutant strain forms a robust biofilm. *cwp84* mutant grown in BHIS + glucose for 72 hours in liquid culture forms macroscopic structures, whereas the 630Δ*erm* parent and the complemented *cwp84* mutant strains do not (Panel A). Insets below the centrifuge tubes are magnified views of the box depicted on the tubes (the scale bar corresponds to 0.1 cm). Panel B shows biofilms of the parental 630Δ*erm* strain, the *cwp84* mutant and the complemented *cwp84* mutant strain depicted before (Bi) or after (Bii) crystal violet staining. Panel C depicts biofilm quantitation. Data are representative of at least three independent experiments, each performed in triplicate. The error bars represent standard deviation. Panel D depicts enumeration of biofilm-associated bacteria generated by the various genotypes shown. Significantly different (*p* < 0.05) ratios are indicated by asterisks (Wilcoxon test for the comparison of Δ*erm* 630/*cwp84* and Student *t* tests for the others).

**Fig 2:**
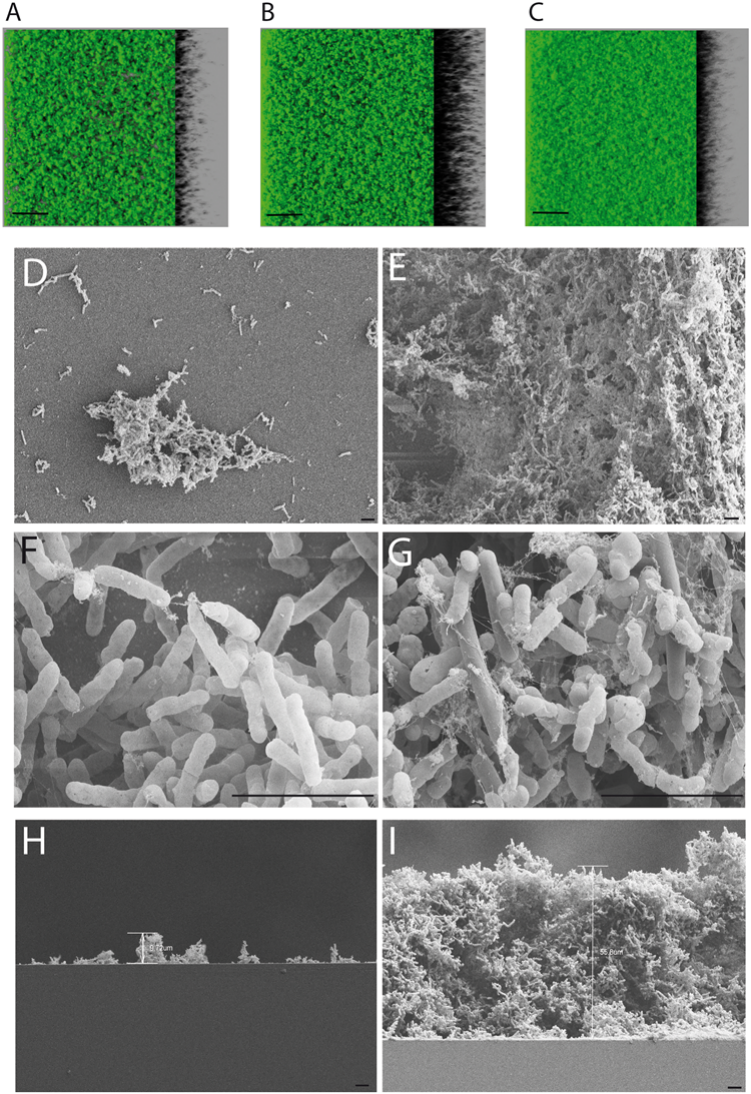
*C*. *difficile* biofilms are visualized by CLSM and electron microscopy. Biofilms elaborated by the 630Δ*erm* parent strain (A) and the cognate *cwp84* mutant and complemented strains (B and C respectively) were visualized by CLSM. The bar corresponds to a height of 50μm, captured via z-axis scans. Biofilm thickness averaged 39.2± 6.9μm for the *cwp84* mutant strain and was significantly higher (*p*<0.01; paired Student *t* test) than that of the parent and complemented strains (17.7±5.8μm and 18.5±2.8 μm respectively). A top-down view using electron microscopy is shown for the parental and *cwp84* mutant strains, in lower magnification (D and E respectively) or in higher magnification (F and G respectively). A side-view is shown for the parental and *cwp84* mutant strains at lower magnification (H and I respectively). The *cwp84* mutant biofilm (60.3±4 μm thick) is significantly larger than the biofilm formed by the parental strain (11.5±1.6 μm) (p<0.001; paired Student *t* test). Black bars, 5μm.

Two reports showed that the *cwp84* mutant has a different behavior of growth compared to the 630Δ*erm* parent strain [9,10]. We observed that the *cwp84* mutant strain grew slower than the parental strain (not shown) and after 48h and 72h formed distinct aggregates and plaque-like structures (Fig 1A). To further investigate the plaque-like phenotype, we performed standard crystal violet-based biofilm assessments of static cultures as previously described [11], and observed intensely-staining aggregates for only the mutant strain (Fig 1B). Data quantitation revealed that the biofilm formed by the *cwp84* mutant strain was 72-fold increased over that of the 630Δ*erm* parent or complemented *cwp84* mutant (Fig 1C). Further, in this biofilm context, *cwp84* biomass (as measured in bacterial counts) was in excess of two orders of magnitude compared to that of the parent or complemented strains (Fig 1D). Sporulation efficiency and spore recovery was not impacted during biofilm formation (3 days) and was low in both parent and mutant strains (<0.05% of spores; data not shown). In order to visualize mature biofilms, both confocal laser scanning microscopy (CLSM) and electronic microscopy were employed (Fig 2). In CLSM, the biofilm of the *cwp84* mutant strain was denser and thicker compared to the biofilms of 630Δ*erm* and the complemented strains. Biofilm thickness averaged 39.2± 6.9μm for the *cwp84* mutant strain and was significantly higher (*p*<0.01; paired Student *t* test) than that of the parent and complemented strains (17.7±5.8μm and 18.5±2.8 μm respectively). Using electron microscopic visualization, at 72 hours, a substantially larger biofilm was formed by the *cwp84* mutant (60.3±4 μm thick) compared with that formed by the parental strain (11.5±1.6 μm) (Fig 2H and 2I). The observed difference is significant (*p*<0.001; paired Student *t* test). Biofilm sizes observed by SEM and confocal microscopy were not directly compared due to differences in the solid surface used in both experiments (see Materials and Methods). Also evident and unique in the mutant was copious elaboration of a fibrous and reticulate network, reminiscent of dehydrated biofilm surface matrix (Fig 2D to 2G).

### Cwp84 proteolytic activity modules biofilm formation

**Fig 3:**
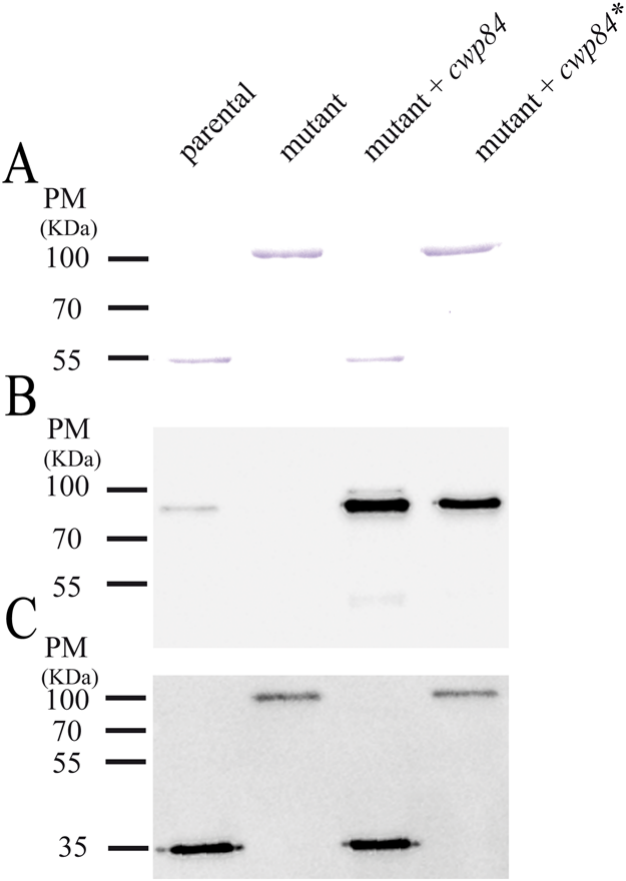
S-layer profiling of *C*. *difficile* strains. 630Δ*erm* (lane 1), *cwp84* mutant (lane 2), *cwp84* mutant+*cwp84* (lane 3), *cwp84* mutant+cwp84* (lane 4) grown in planktonic culture before protein extraction. S-layer proteins were analyzed using SDS-PAGE (Panel A) or via immunoblots using antisera specifically directed against Cwp84 (Panel B) or the LMW-SlpA P36 (Panel C). Equivalent amounts of proteins were loaded in each well.

To assess whether the phenotypes observed above were due to the absence of the Cwp84 protein *per se*, or the absence of its proteolytic activity, we engineered a non-functional recombinant molecule (Cwp84*), replacing two of the three amino acids of the protein’s catalytic triad (C116 and H262), with an alanine residue. Cwp84* was expressed in the *cwp84* mutant strain, and surface-layer extracts analyzed using antisera specifically directed against Cwp84 or SlpA. As expected, in the *cwp84* mutant, and in contrast to parent and complemented strains, Cwp84 protein was not detected, and SlpA was not cleaved (Fig 3). In the strain expressing the catalytically-deficient protease, Cwp84* was indeed expressed, but SlpA was not cleaved (Fig 3B and 3C). Further, the expression of Cwp84* in the *cwp84* mutant was not able to restore the parental biofilm phenotype (Fig 1C), thus implicating the absence of Cwp84 proteolytic activity as being required for biofilm formation.

### Biofilm production does not directly correlate with SlpA sequence

**Fig 4:**
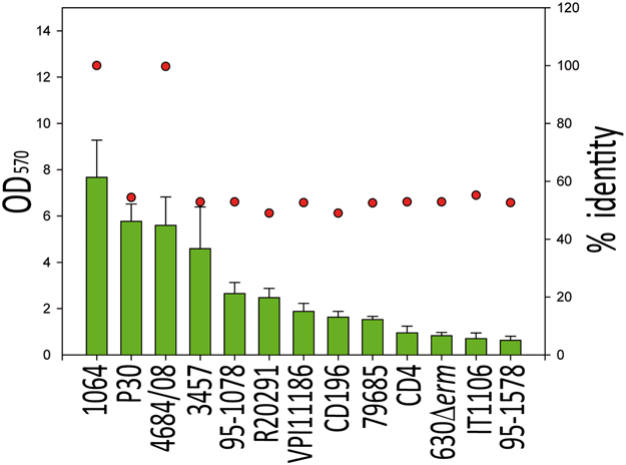
Biofilm-forming ability is independent of SlpA primary sequence. Biofilm propensity of different strains of *C*. *difficile* (X-axis) was compared using crystal violet staining (Y-axis; filled green bars). SlpA primary amino acid sequence was also determined for these same strains, and percentage identity (filled red dot) compared with that of the 1064 comparator strain. The strains evaluated were P30, 4684/08, 3457, 95–1078, R20291, VPI11186, CD196, 79685, CD4, 630Δ*erm*, IT1106 and 95–1578 (see Table 1 for details). Biofilms formed by 1064, P30, 4684/08 and 3457 (group 1, OD_570_>4.6), 95–1078, R20291, VPI11186, CD196 and 79685 (group 2; 1.5 <OD_570_< 2.65) and CD4, 630Δ*erm*, IT1106 and 96–1578 (group 3; OD_570_< 0.95) are significantly different (*p*<0.01; Student *t* test). However, amino acid sequences between groups are not significantly different.

Since Cwp84 processes SlpA, and, as demonstrated above, that its proteolytic activity impacts biofilm formation, it is formally possible that the *cwp84* mutant phenotype is manifested via uncleaved SlpA, suggesting that SlpA may be of importance in the biofilm formation in *C*. *difficile*. To date, *slpA* has not been disrupted in *C*. *difficile*, likely because it is essential [17]. Despite multiple attempts, we were also not able to inactivate it (data not shown). Therefore, to investigate if SlpA impacts biofilm formation in *C*. *difficile*, 13 strains expressing different *slpA* alleles were tested for their biofilm-forming abilities (Fig 4). The 1064, P30, 4684/08 and 3457 strains produced robust biofilms, whereas CD4, 630, IT1106 and 96–1578 were weak biofilm producers. Three strains forming the most robust biofilms belong to the ribotype 014/020 (Table 1). However, SlpA from the P30 strain is different from the 1064 and the 4684 strains, suggesting that the ability to form a biofilm is not strictly due to SlpA primary amino acid sequence. Overall, no correlation was evident between the SlpA amino acid sequence and the ability of the strains to produce biofilms (Fig 4).

**Table 1:** Ribotypes and SlpA accession numbers (Genbank, http://www.ncbi.nlm.nih.gov/genbank) used in the study.

Strain	Ribotype	SlpA accession number
1064	014/020	HF569017
P30	014/020	KM104169
4684/08	014/020	AAZ05984
3457	other*	KM099225
95–1078	other	KM099223
R20291	027	C9YQ17
VPI11186	other	KM099227
CD196	027	C9XP98
79685	other	KM104169
CD4	053-like	KM099226
630D*erm*	012	Q183M8
IT1106	078	AAZ05994
95–1578	012	KM099224

* The ribotype “other” corresponds to a ribotype that is not 070, 078/126, 002, 012, 029, 053, 075, 005, 018, 106, 131, 117, 003, 019, 046, 050, 014/020/077, 001, 015, 017, 023, 027, 056, 081 or 087.

### Toxin, stress and surface protein abundance is altered in the *cwp84* mutant biofilm

**Fig 5:**
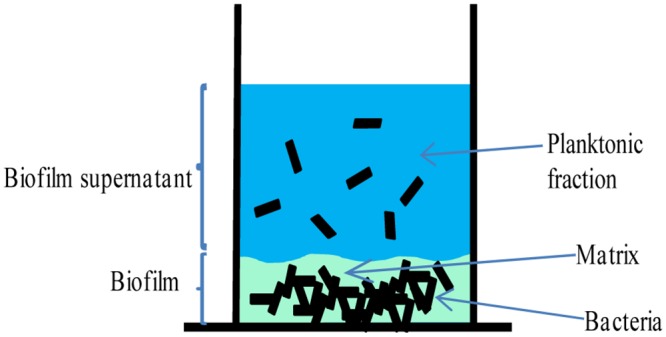
*C*. *difficile* biofilm development. Schematic representation of the *C*. *difficile* plate-grown biofilm. Matrix-associated and supernatant (planktonic) bacteria are shown. Specific individual biofilm fractions were harvested for proteomics as described in the Methods.

Due to the vastly different architecture of the parental and *cwp84* mutant biofilms, and previously identified alterations in surface protein composition of the *cwp84* mutant [9], we performed proteomic analysis on three fractions prepared from bacterial biofilms: the surface proteome of biofilm-associated bacteria, the biofilm matrix fraction, and the biofilm supernatant (Fig 5). This latter compartment contained proteins released from both the biofilm as well as planktonic bacteria growing outside the biofilm or released from the biofilm. All detected proteins are presented in S1 Table, S2 Table and S3 Table. Among these, distribution of surface, matrix and released proteins of the 630Δ*erm* are presented Table 2. Both parental and *cwp84* mutant strains were assayed and compared in Tables 3 and 4. In the biofilm formed by the 630Δ*erm* parental strain, ~33% of identified molecules were Cwp proteins (17 out of 59). Except for Cwp13 and Cwp22, most of the Cwp proteins were found in the biofilm matrix and/or in the biofilm supernatant. Moreover, Cwp2, Cwp6, Cwp12, Cwp16, Cwp19, Cwp25 and CwpV were found mostly in the supernatant fraction. Interestingly, we observed accumulation of the glucosyltransferase toxin TcdA as well as the putative iron transporter FeoB1 (CD630_14790) in the biofilm matrix fraction. Overall, we detected up to 10 proteins putatively involved in the cell wall modeling (such as CD630_01830) and up to 4 involved in the stress response (such as CD630_08270) that were concentrated in one biofilm compartment, namely, the cell surface, the matrix, or the supernatant (Table 2). Comparison of the above results with those obtained for the *cwp84* mutant strain revealed some specific differences (Tables 3 and 4). In general, the proteomes of bacterial surface or released proteins were largely similar between parental and mutant strains except for a few molecules (Cwp2, Cwp6 or CD630_18980). SlpA was found to be increased in abundance in the *cwp84* mutant biofilm matrix. In contrast, Cwp16 and Cwp17 were only detected in the matrix of the parental strain. In all, and using the stringent analysis (see Materials and Methods) that we employed for this study, only six bacterial surface-associated proteins were quantitatively significantly different between the *cwp84* mutant and parental strains, and seven proteins were similarly changed in quantity in the biofilm supernatant. Taken together, these results suggest that alterations in abundance of only a few proteins significantly impacts biofilm properties of the *cwp84* mutant.

**Table 2:** Distribution of surface, matrix and released (secretome) proteins of the 630Δ*erm* parental strain.

**630**Δ***erm* surface proteome of bacteria in biofilm**
CD630_24980 DacF*
CD630_11350 Putative SH3-domain protein*
CD630_02690 FlgG
CD630_05540 Sip2
CD630_15220 Putative polysaccharide deacetylase
CD630_17510 Cpw13*
**630**Δ***erm* matrix**
D630_02370 FliD
CD630_06630 TcdA
CD630_07810 Putative penicillin-binding protein
CD630_08280 Putative oxidative stress glutamate synthase
CD630_08740 ABC-type transport system, sugar-family
CD630_08770 ABC-type transport system, sugar-family
CD630_11840 FabF
CD630_14790 FeoB1
CD630_14840 SsuA
CD630_18220 Bcp
CD630_22690 FruABC
CD630_23880 Putative peptidoglycan-binding/hydrolysing
CD630_26670 PtsG-BC
CD630_27350 Cwp14
CD630_27840 Cwp6
CD630_27860 Cwp5
CD630_27980 Cwp9
CD630_28010 Conserved hypothetical protein
CD630_30300 PTS system, glucose-like IIBC component
CD630_31920 Cwp21
CD630_32840 Serine protease, HrtA family
CD630_34680 AtpD
**630**Δ***erm* biofilm secretome**
CD630_24980 DacF*
CD630_11350 Putative SH3-domain protein*
CD630_01830 Putative cell wall hydrolase
CD630_02550 FlgE
CD630_05140 CwpV
CD630_05880 Conserved hypothetical protein
CD630_08270 Rbo
CD630_08440 Cwp25
CD630_08550 OppA
CD630_10350 Cwp16
CD630_10470 Cwp18
CD630_13040 Acd
CD630_17510 Cpw13*
CD630_21270 Putative exported protein
CD630_24020 Putative cell wall hydrolase; phosphatase-associated protein
CD630_26720 AppA
CD630_27130 Cwp22
CD630_27670 Cwp19
CD630_27680 Putative cell-wall hydrolase
CD630_27890 Cwp66
CD630_27910 Cwp2
CD630_27940 Cwp12
CD630_27950 Cwp11
CD630_35590 FtsH2
CD630_36690 Putative exported protein

* proteins found in the same quantity in the surface proteome of bacteria in biofilm and in biofilm secretome.

**Table 3:** Proteins increased in abundance in the *cwp84* mutant biofilm proteome compared with the 630Δ*erm* parental strain biofilm proteome.

***cwp84* mutant surface proteome of bacteria in biofilm**
CD630_08730 ABC-type transport system, sugar-family, extracellular solute-binding protein
CD630_02390 FliC
CD630_07550 Putative cell surface protein
CD630_27910 Cwp2
***cwp84* mutant matrix**
CD630_08280 Putative oxidative stress glutamate synthase
CD630_08730 ABC-type transport system, sugar-family, extracellular solute-binding protein
CD630_16230 Putative oxidoreductase
CD630_02550 FlgE
CD630_08270 Rbo
CD630_08550 OppA
CD630_02390 FliC
CD630_26720 AppA
CD630_27840 Cwp6
CD630_27910 Cwp2
CD630_27930 SlpA
CD630_34680 AtpD
CD630_36690 Putative exported protein
***cwp84 mutant* biofilm secretome**
CD630_08280 Putative oxidative stress glutamate synthase
CD630_18980 Putative phage-related cell wall hydrolase (endolysin)
CD630_06630 TcdA
CD630_05140 CwpV
CD630_27130 Cwp22

**Table 4:** Proteins increased in abundance in the 630Δ*erm* parental strain biofilm proteome compared with the cwp84 mutant biofilm proteome.

**630**Δ***erm* surface proteome of bacteria in biofilm**
CD630_26720 AppA
CD630_32840 Serine protease, HrtA family
**630**Δ***erm* matrix**
CD630_21760 ABC-type transport system, cystine/aminoacid-family permease
CD630_10350 Cwp16
CD630_10360 Cwp17
**630**Δ**erm biofilm secretome**
CD630_08270 Rbo
CD630_28010 Conserved hypothetical protein

### *cwp84* mutant bacterial surface is more hydrophobic than the parental strain, but adhesion properties are unchanged

**Fig 6:**
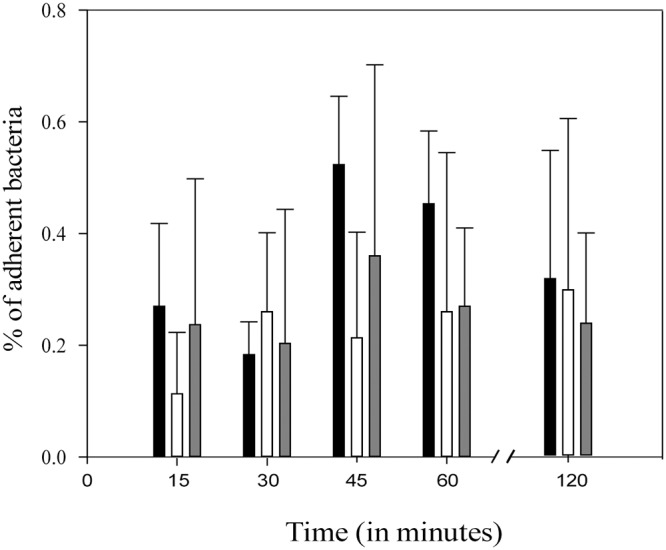
Parental and *cwp84* mutant strains display similar adhesion to abiotic surfaces. Percentage initial adhesion of 630Δ*erm* (black bars), *cwp84* mutant (white bars), *cwp84* mutant +*cwp84* (grey bars) strains to a polypropylene matrix for 15–120 minutes. Error bars represent standard deviation. Data are representative of four independent experiments each performed in triplicate. The observed differences are not significant (Student *t* test).

We assessed cell-surface biophysical impact on the *cwp84* mutant since the surface proteome differed from that of the parental strain, SlpA was not cleaved, and specific proteins predicted to be involved in cell-surface remodeling (CD630_18980; cell-wall hydrolase) were differentially abundant. Standard bacterial hydrophobicity assays were performed using the alkane hydrocarbon hexadecane. The parental 630Δ*erm* strain as well as the complemented *cwp84* mutant were weakly hydrophobic (3.91% ± 0.6% and 1.02% ±0.96% respectively), whereas the *cwp84* mutant was significantly highly hydrophobic (44.27% ± 4.59; *p*<0.005; paired Student *t* test) in comparison with the parental strain. In other species, such as *Lactococcus lactis*, adhesion correlates with hydrophobicity [18]. We therefore assessed if altered hydrophobicity impacted bacterial adherence to abiotic surfaces, theorizing that initial attachment may have downstream consequences on biofilm formation. Parental, mutant and the complemented strains were analyzed. Initial adhesion levels were very low (ranging from 0.1%- 0.5%), and no significant differences were observed between strains (Fig 6) suggesting that Cwp84 was not involved in initial adhesion of *C*. *difficile* on the tested surface, the first biofilm step.

### Growth of the *cwp84* mutant strain is less impacted when it forms a biofilm

**Fig 7:**
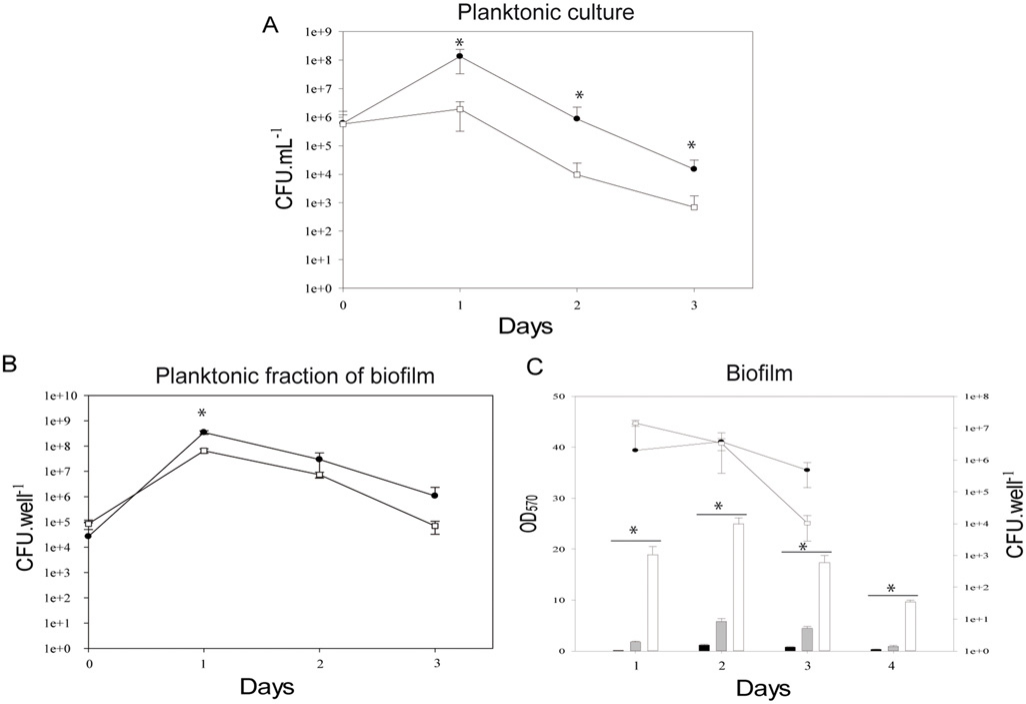
*cwp84* mutant displays a growth defect only in planktonic culture. The 630Δ*erm* (●) and *cwp84* mutant (□) strains were grown in competition in agitated planktonic culture (Panel A) or recovered from biofilm supernatants (non-settled/escaped bacteria; Panel B), and colony forming units (CFU) enumerated in three independent experiments. The planktonic cultures of both strains are significantly different (*p*<0.05; Student *t* test) whereas there is no significant difference for bacteria grown in biofilm, except for bacteria from the biofilm supernatant at 24h. Panel C, for biofilm-associated bacteria, colony counts (CFU), biomass after competition (grey bars, in vitro competition 630Δ*erm* and *cwp84* mutant strains) or biomass after individual growth (630Δ*erm*, black bars; *cwp84* mutant, white bars) were determined. Error bars represent standard deviation of the mean. Biofilms of strains in competition were significantly different to those formed by individual strains (*p* <0.05; Student *t* test). Significantly different ratios are indicated by asterisks.

The *cwp84* mutant strain grew slower ([9], S3 Fig and see below), but formed a thicker biofilm and grew better under biofilm conditions than the 630Δ*erm* parental strain (Figs 1 and 2). We therefore tested the growth of both strains in competition in planktonic culture and biofilm conditions. In planktonic culture, the mutant exhibited a significant growth defect (two orders of magnitude) compared to the parental or complemented strains (Fig 7A). This defect continued to manifest in a competition setting between parental and mutant strains in the planktonic fraction of biofilm (Fig 7B). However, when parental and mutant strains were tested together in competition in the biofilm state, bacterial recovery was initially similar for both strains (up to 48 hours), but the *cwp84* mutant strain was progressively eliminated thereafter, along with resulting biofilm destabilization (Fig 7C).

### The *cwp84* mutant strain proliferates in the axenic murine GI tract, and recapitulates in vitro dynamics

**Fig 8:**
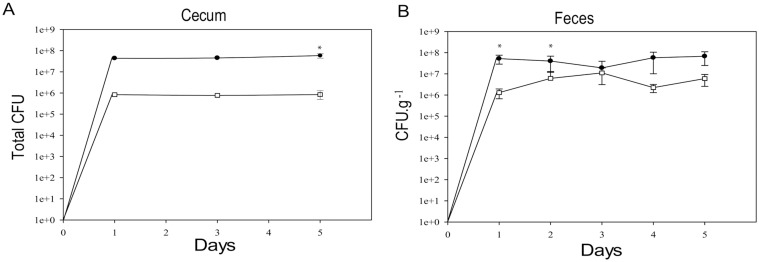
In vivo competition of parental and *cwp84* strains. The 630Δ*erm* parent strain (●) and *cwp84* mutant (□) were grown in competition in axenic mice for up to 5 days. In vivo analyses of bacterial counts were performed using feces or via cecal enumeration as described in the Methods. Error bars represent standard deviation. Significantly different (*p* < 0.05, Student *t* test) ratios are indicated by asterisks.

Since the *cwp84* mutant strain grew better in the biofilm state, (Fig 7), we investigated whether this phenotype may be beneficial in vivo. In order to eliminate commensal microbiota influences for this preliminary assessment, a murine *C*. *difficile* dixenic model was used. Mice were infected with an approximately 1:1 mixture of both genotypes, and bacterial burden enumerated via both cecal content as well as fecal pellet plating (Fig 8). Both strains, as already observed in this model [19], proliferated in the mice, with the parental strain showing an increased burden (2 orders of magnitude) over the mutant, reminiscent of our in vitro competition results. In the cecum, the relative amounts of both strains remained constant over the study period; however, this was not reflected in the fecal contents, where the mutant strain recovery varied over time.

## Discussion

*C*. *difficile* biofilms have only recently been identified, and are yet to be thoroughly characterized. With the exceptions of *luxS* and *cwp84*, all other *C*. *difficile* genes evaluated to date have no, or very modest, effects on biofilm formation [11,12]. Even the *spo0A* mutant was still able to form a biofilm (although weaker than the parental strain), unlike in *B*. *subtilis* wherein the *spo0A* mutant was required to form a biofilm [20]. In our study, we observed that the *cwp84* allele in the 630Δ*erm* strain negatively modulates biofilm development, and that the mutant phenotype was fully complemented by providing the gene *in trans*. The mutant we used [9] was engineered via insertional inactivation, unlike that generated by Dapa *et al*, who utilized allelic-exchange methodology to delete the 3’-end of the same gene in the epidemic-associated *C*. *difficile* isolate R20291. Despite strain and manipulation differences, both mutants behave identically under the conditions we describe for biofilm assessment (Fig 1 and S1 Fig). Further, all the biofilm-associated biology we observed is solely due to Cwp84, and does not involve polarity effects, since complementation with just the single gene restored the parental phenotype.

The *cwp84* mutant harbors a growth defect, at least during the exponential phase of growth (Fig 7 and S3 Fig). However after 72h, the relative growth of the *cwp84* mutant is higher than the parental strain (S3 Fig). In parallel, the sporulation, in vitro and in vivo of the *cwp84* mutant is lower than the parental strain (S2 Fig and S3 Fig). In vivo the rate is close to 1% of the total CFU (spores and bacteria). As mentioned, sporulation in *Bacillus* species is linked to the biofilm formation. However, our results suggest that sporulation is only partially linked to biofilm formation for *C*. *difficile*.

The molecular mechanisms underlying the increased biofilm formation of the *cwp84* mutant strain remain to be defined, but two possibilities are highlighted by this study. One involves abundance of cleaved SlpA, and the other invokes a novel role for Cwp84. The former is highlighted by the observation that heterologous expression of *cwp84* in the mutant background restores both cleaved SlpA products as well as the parental biofilm and hydrophobicity phenotypes. However, when SlpA is overexpressed in the parental strain, no change in biofilm was observed (not shown). Further, no correlation was observed between the strain capacity to form a biofilm and corresponding SlpA amino acid sequence. Taken together, these data suggest that SlpA’s role on *C*. *difficile* biofilm formation still remains to be elucidated. However, since uncleaved SlpA accumulates in the biofilm matrix of the *cwp84* mutant, it may ensue that matrix biophysical properties may be sufficiently altered to promote biofilm formation. Indeed, uncleaved SlpA interacts with the bacterial surface and therefore may enhance the bacterial interactions in biofilm through its position in the matrix in the *cwp84* mutant. Alternatively, Cwp84 may be involved independently of SlpA in the observed phenotype by cleaving one or several other bacterial proteins.

In the *cwp84* mutant, protein abundance within the biofilm is altered compared with that of the parental strain. The remodeling of the bacterial surface in the *cwp84* mutant has already been described [9]. Indeed, Cwp66 and Cwp2 were found to be released into the culture supernatant of planktonic bacteria in the *cwp84* mutant strain whereas they were not in the parental strain. However, in the biofilm context, these two proteins were distributed differently. In the parental strain, Cwp2 was mainly found in the supernatant, whereas Cwp66 was localized to the matrix. In the *cwp84* mutant, Cwp2 was found to be accumulated on the bacterial surface and matrix of the *cwp84* mutant in comparison with the parental strain. Thus, the changes in the proteomes of parental and mutant strains in the biofilm, although not numerically predominant, were more than sufficient to result in significant phenotype differences. It is noteworthy that TcdA was observed in the biofilm grown in the presence of glucose. This unexpected production of TcdA may be the result of particular metabolism due to the biofilm state of growth or the glucose total consumption by bacteria after 72h of incubation.

As visualized by microscopy, the architecture of the *cwp84* mutant biofilm is substantially different from that formed by the parental strain (Fig 2). The *cwp84* mutant elaborates a larger and denser biofilm that may also express a thicker matrix. This matrix may contain polymerized proteins, glycans or nucleic acids; some of which may be released from lysed bacteria within the structure. Indeed, Dawson *et*. *al* reported that biofilm matrices contain DNA [12]. Considering the vast hydrophobicity differences we observed, a polysaccharide contribution to the matrix cannot be excluded.

In our *in vivo* studies, both parental and mutant (yet at a lower level in the competition assay) strains proliferated in the murine gastro-intestinal (GI) tract, and maintained consistent bacterial loads over time in the cecum. The mutant strain, from day 3 to day 5 was found at the same level than the parental strain in the feces (non significant difference; Student *t* test). This suggests that there may be stable GI tract reservoirs of bacteria, which cannot be appreciated solely by estimating fecal carriage. These reservoirs may ultimately transition into the biofilm state although it is unlikely that this would happen over the short time-scale corresponding to fulminant CDI manifestation, and especially in the context of a suppressed, but still viable microbiota. However, it is possible that in the context of relapsing disease, pre-existing populations of *C*. *difficile* bacteria may enter and colonize the biofilm state more readily over time. Indeed, it has been reported that *C*. *difficile* spores preferentially localize to biofilm-rich regions of a chemostat-based human gut model [21]. The cascade of events resulting in a robust biofilm may be controlled by factors like Cwp84, which, via their negative modulatory role as shown in this study, may serve to keep some bacteria in the planktonic state so that the infectious and intoxication processes may proceed unchecked.

## Materials and Methods

### Bacterial strains, plasmids and growth conditions

*Escherichia coli* strains were grown at 37°C in LB (Luria Broth, Difco Laboratories) [22]. *E*. *coli* TG1 was used as the host for plasmid constructions. *E*. *coli* HB101 (pRK24) was used for mating experiments. The following antibiotics (Eurobio) were added to the culture medium: kanamycin (40 mg L^-1^), ampicillin (100 mg L^-1^), chloramphenicol (12.5 or 25 mg L^-1^). *C*. *difficile* strains, 630Δ*erm* [23],NF2184 (the *cwp84* mutant strain, [Er^R^]) [9] and the NF2184 containing the pMTL960Ω*cwp84* or the pVP75 plasmids, were grown at 37°C in Brain Heart Infusion (BHI) or BHIS+ glucose (BHI supplemented with Yeast Extract (5mg.ml^-1^, Difco), cysteine (0.1%; Fisher) and glucose (0.1 M; Sigma)). The following antibiotics were added to the culture medium: thiamphenicol (15 mg L^-1^, MP Biomedical), supplement (composed of 250 mg L^-1^ of D-cycloserine and of 8 mg mL^-1^ of cefoxitin, Oxoid), and erythromycin (5 mg L^-1^, sigma-aldrich). *C*. *difficile* strains were grown in anaerobic condition (90% N_2_, 5% CO_2_ and 5% H_2_; Messer) in anaerobic chamber. The CD1064, P30, 4684/08, 3457, 95–1078, R20291, VPI11186, CD196, 79685, CD4, IT1106 and 95–1578 come from our lab collection [19]. Ribotype analysis was performed by “CNR Bactéries anaérobies et botulisme (*Clostridium difficile*)—Laboratoire Associé” (Hopital Saint-Antoine, Paris) using the method described by Stubbs *et al*. [24].

### Mutagenesis and cloning of *cwp84* gene

Site-directed mutagenesis was performed as previously described [8] using the plasmid pET28a(+)Ωcwp84_C116A_ and the oligonucleotides 5’-GAGGGCTCCATTAAATGCCGCTGTAGCGATAGTAG-3’, and 5’-CTACTATCGCTACAGCGGCATTTAATGGAGCCCTC-3’. The resulting plasmid (pTC60) harbors the *cwp84* gene encoding the modified Cwp84 where both the C116 and the H262 were replaced by an alanine residue. To express the inactive Cwp84 in *C*. *difficile*, the corresponding gene was cloned as follows. An Overlap Extension PCR cloning [25] was performed using the oligonucleotides VP98 (GGATCCgtctctttatgtttttttaaaatcaaatataatc), VP99 (TACTCCATCTAGAGTTTTATGGTTTTCTGCtgagacaggtattgttgacactattaaaaagc) and 630 genomic DNA as template and VP100 (GCTTTTTAATAGTGTCAACAATACCTGTCTCAgcagaaaaccataaaactctagatggagta), VP101 (GAATTCctattttcctaaaagagtatttagttcattaaaagc) and pTC60 as template. The resulting PCR DNA fragment was cloned into the pCR-Blunt vector (Invitrogen) and the resulting construct named pVP73. pVP73 was retricted using *Kpn*I/ *Xho*I, and the sub-cloned into similarly digested pMTL84151 plasmid to generate pVP75. pVP75 was transferred from *E*. *coli* HB101 (pRK24) to *C*. *difficile* 630 by conjugation as described [26]. Complementation of the *cwp84* mutant was performed using the pMTL960Ω*cwp84* plasmid [9].

### Biofilm assay

The biofilm assay was performed in the 24-well polystyrene plates (Costar, USA). A 16h culture of *C*. *difficile* strains was diluted (1/100) into fresh BHIS containing 1.8% glucose (Sigma). 1ml of the culture was placed into each well. The plate was incubated under anaerobic conditions (90% N_2_, 5% CO_2_ and 5% H_2_) at 37°C for 3 days. To prevent evaporation of liquid, 1 mL of PBS was placed in empty wells. After 3 days, the plate was gently washed twice with 1.5 mL of sterile PBS. The plate was incubated at 37°C for 10 minutes. One mL of 0.2% crystal violet was introduced into each well, and the plates incubated for 30 minutes at 37°C. The wells were then gently washed twice with 2mL sterile PBS, the incorporated crystal violet dye extracted with 1ml of alcohol-acetone (80%/20%), and its absorbance values determined at 570 nm. Three wells per plate were incubated with medium only for background staining quantitation, and this value was subtracted from sample biofilm values measured after staining.

### Adhesion assays

In order to evaluate bacterial adhesion (the first step of biofilm formation), the following assay was performed. Adhesion assays were performed in 24-well polystyrene plates (Costar, USA). A 16 hour culture of *C*. *difficile* was diluted (1/100) into fresh BHIS containing 1.8% glucose and 1 mL was introduced into each well. The concentration of spores was negligible (less than 0.03% of the total). The plate was incubated under anaerobic conditions at 37°C. After various contact times (from 15 min to 120 min), non-adherent (planktonic) bacteria were removed. The wells were then washed twice with1.5 mL PBS. 1mL of PBS was finally added to each well, and adherent bacteria were removed by scraping, and enumerated by plating on BHI-yeast supplemented with blood (5%). The percentage of adherent bacteria at each time point of the experiment was obtained using the following formula: (N_a_/N_T_)*100, where N_a_ is the number of adherent bacteria and N_T_ is the number of total bacteria in the well (adherent and planktonic bacteria). The 1.5 mL PBS washes were not included in the assessment of the N_T_. Noteworthy, after two hour incubation, a doubling of the bacterial population (taking in account in the percentage calculation) was observed for all tested strains (data not shown).

### CLSM analysis of biofilm formation

Three-dimensional structure of 3-day-old biofilm was analyzed by scanning confocal laser microscopy. Biofilms were grown in the 24-wells polystyrene plates as described in the biofilm formation assay. Cells of 3-days biofilms were fluorescently stained with the cells permeate nucleic acids SYTO 9 dye at 5μM (Molecular probes, Life Technologies). After 20 min of incubation in the dark to enable fluorescent labeling of the bacteria, the plates were then mounted on the motorized stage of the confocal microscope. The observations were carried out at the INRA MIMA2 imaging center with a SP2 AOBS confocal laser scanning microscope (LEICA Microsystems, France). The microtiter plates were scanned using a 63×/1.4 N.A. oil immersion objective lens using an excitation wavelength of 488 nm (argon laser, 30% intensity), with emission wavelengths collected from 480 to 530 nm for the green emitted SYTO9 fluorescence. Three-dimensional projections of biofilm structures were reconstructed using the Easy 3D function of the IMARIS software (Bitplane, Switzerland) directly from xyz images series. Biofilm thickness (μm) was directly measured from xyz stacks.

### Electron microscopy

To further visualize *C*. *difficile* biofilm structure, Field Emission Scanning Electron Microscopy (FESEM) was performed as previously described [27]. Briefly, bacterial biofilms were grown as described above, but on glass cover-slips, fixed in a solution of 1% glutaraldehyde in 0.1 M sodium cacodylate (Santa Cruz Biotech, Santa Cruz, CA) for 1 hour, washed with water, stained with 1% osmium tetroxide (Electron Microscopy Sciences, Hatfield, PA), and then dehydrated in a series of ethanol washes. The fixed specimens were mounted, coated in platinum, and imaged using a Hitachi S-4800 FESEM instrument.

### *C*. *difficile* planktonic and biofilm protein extractions

S-layer proteins (SLPs) were prepared by the low-pH glycine extraction method as previously described [28]. For biofilm, the supernatant was removed carefully. Before protein extraction, biofilms were washed at least twice in order to keep only firmly attached bacteria constituting the biofilm and remove contaminant material. For protein extraction from biofilms, 230 and 23 wells worth of biofilm material were pooled respectively from the 630Δ*erm* and the *cwp84* mutant. Surface-associated proteins were extracted using the method of Wexler et al [29] with some modifications. Bacteria were removed by centrifugation (5,000 g, 20 min, 4°C), and the supernatant filtered by passage through a 0.45 μm sterile filter. The proteins in the supernatant were precipitated by adding 10% trichloroacetic acid (Sigma) at 4°C for 4 hours, and pelleted by centrifugation (7,000 g, 60 min, 4°C). The pellet was washed 3 times with of 96% ethanol, placed at 37°C for 10 minutes for alcohol evaporation, and 100 μL of 8 M urea was added. Matrix proteins were extracted by resuspending biofilms in 10 mL of PBS followed by centrifugation (15,000g 10 min, 20°C). The supernatant was then treated in the same manner as that described for harvesting biofilm supernatant molecules as described above.

### Comparative proteomics

Both Orbitrap (non-quantitative) and iTRAQ-based proteomics (isobaric tagging for relative and absolute quantitation; fully quantitative) were employed for this study. Sample preparation for proteomics was performed as described previously [27]. Briefly, approximately 100μg of protein from each sample was denatured in SDS, reduced in Tris-(2-carboxyethyl) phosphine, and alkylated in iodoacetamide. Following trypsin digestion (ratio of 1:10), iTRAQ reagent labeling was performed according to the iTRAQ kit instructions (AB SCIEX, Framingham, MA). Strong cationic exchange (SCX) fractionation was then performed on a passivated Waters 600E HPLC system, and 15 SCX fractions collected and resuspended in an acetonitrile/trifluoroacetic acid solution. Each fraction was autoinjected onto a Chromolith CapRod column (150 X 0.1 mm, Merck), separated over a solvent gradient, and automatically spotted onto a stainless steel MALDI target plate every 6 seconds (0.6 μl per spot), for a total of 370 spots per original SCX fraction, which were analyzed on an ABI 4800 MALDI TOF-TOF mass spectrometer (AB SCIEX, Framingham, MA). Protein identification and quantitation was performed using the Paragon algorithm implemented in Protein Pilot 3.0 software against the *C*. *difficile* strain 630 protein database plus common contaminants. Using a technical replicate and standard sensitivity curve analyses [30], fold change cutoffs were calculated assuming a false discovery rate (FDR) of 10%. Further, and for each identified protein, hypergeometric testing was also performed to calculate a *p*-value of the difference in expression of each test protein compared to the same protein in the control dataset (values ≤0.05 were accepted). Only when both FDR and p-value criteria were satisfied was a protein deemed to be differentially abundant. Protein functional classification was performed using RAST (Rapid Annotation using Subsystem Technology; [31]).

### Immunodetection

The protein preparations from the different extracts were quantified and equivalent amounts loaded on a 12% polyacrylamide SDS gel. For the western blot, proteins were transferred to polyvinylidene difluoride membrane (Amersham Biosciences). For the dot blot analysis, anti-Cwp84 [7] or anti-p36 (to detect SlpA) [32] antibodies were diluted at 1:10,000, and immunoreactivity visualized using an anti-rabbit secondary antiserum conjugated to horseradish peroxidase (1:2,000; Sigma), a chemiluminescent HPR substrate (Millipore) and the imaging system Fusion Fx (Vilber Lourmat).

### Hydrophobicity assays

Hydrophobicity was tested with hexadecane by the method of Bellon-Fontaine *et al*. [33] using technical duplicates. The absorbance at 405nm of the suspensions (A_0_) was measured using a spectrophotometer (S.250, Secomam). Each bacterial suspension (2.4 mL) was vortexed for 2 minutes with 0.4 ml n-hexadecane (Sigma). The mixture was allowed to stand for 15 minutes to ensure complete separation of the two phases. The absorbance of the water phase A was then measured. The percentage of hydrophobic properties was subsequently calculated by the following equation: % hydrophobicity = ((A_0_-A)/A_0_)*100. Four tests were performed on each sample. Significance was calculated using the Pearson test.

### In vitro competition assays

Planktonic and the biofilm bacteria competitions were performed in independent experiments. Planktonic cultures were agitated during the 72 hour incubation using a magnetic barrel to avoid formation of aggregates and biofilm. After 16 hours, the absorbance of each culture was measured and adjusted. Then the cultures were diluted 1:100, mixed, incubated anaerobically for 72h hours and then enumerated. At day 0, the strains were adjusted according to their OD_600_, mixed and directly enumerated by plating. Survival was monitored by bacterial enumeration on BHI or BHI/erythromycin agar to differentiate the strains. For biofilm competition, 3 wells for each ratio above were stained using crystal violet as described above.

### In vivo competition assays

*In vivo* experiments were performed at the Central Animal Care facility of the Faculty of Pharmacie, University Paris-Sud. Six week old female axenic C3HAx mice were obtained from CNRS Orléans, France. All animal studies were conducted according to European Union guidelines for the handling of laboratory animals (http://ec.europa.eu/environment/chemicals/lab_animals/home_en.htm) and procedures for infection, euthanasia and specimen collection were approved by the Ethics Committee CAPSUD (Protocol 2012–111). Mice do not receive any antibiotic beforehand. During the experiment, no symptoms were observed. A mix of 630Δ*erm* bacilli (1.4X10^6^ CFU.mL^-1^) and *cwp84* mutant (4.09X10^5^ CFU.mL^-1^) strains were administrated to 7 mice. Feces were analyzed every days. 2 mice were euthanized by cervical dislocation on Days 1 and 3. Three mice were euthanized on Day 5. *C*. *difficile* burden in the ceca of each mouse was analyzed by selective plating on BHI agar as described above.

### Statistical analysis

Statistical analyses were performed using a standard software package and Wilcoxon tests to assess significance (if the sample number was equal to or above four), or the Student *t* test (if the sample number was equal to three).

## Supporting Information

S1 Fig *cwp84* mutants obtained by the allelic exchange method in the 630Δ*erm* and the R20291 strains harbor the same phenotype than the *cwp84* mutant obtained by the clostron method in the 630Δ*erm* strain. Biofilms of R20291, R20291Δ*cwp84* (CRG2549), R20291Δ*cwp84*+*cwp84* (CRG3059) [11], 630Δ*erm*, 630Δ*erm*Δ*cwp84* (CRG2302) and 630Δ*erm*Δ*cwp84*+*cwp84* (CRG2445) [34] strains were quantified using violet crystal. Data are the average of two independent experiments, each performed in technical triplicates.
(DOCX)

S2 Fig In vivo competition of parental and *cwp84* strains, bacilli and spores details. The 630Δ*erm* versus *cwp84* mutant were grown in competition in axenic mice for up to 5 days. In vivo analyses of bacterial (630Δ*erm* in blue, *cwp84* mutant in green) and spores (630Δ*erm* in pink, *cwp84* mutant in red)counts were performed using feces (A) or via cecal (B) enumeration as described in the Methods. Error bars represent standard deviation. CFUs counts were performed as described in the material and methods, except that the sample was used before and after a 70°C incubation for 30 minutes to differentiate between bacteria and spores as described in Burns and Minton 2011 [35].
(DOCX)

S3 Fig 630Δ*erm* and *cwp84* mutant kinetics over 72h of growth. The 630Δ*erm* (blue curves) and *cwp84* mutant (red curves) strains were grown separately in agitated planktonic culture. Colony forming units (CFU) were enumerated in three independent experiments and the content of spores and bacilli is presented in the Panel A and B, respectively. Spores and bacilli counts were performed as described in S2 Fig.
(DOCX)

S1 Table protein rations in the proteomes of the 630Δ*erm* and the *cwp84* mutant strain. (XLSX)

S2 Table iTRAQ-based proteomics. (XLSX)

S3 Table Orbitrap-based (non-quantitative) proteomic. (XLSX)

## References

[1] Hall-Stoodley L, Costerton JW, Stoodley P (2004) Bacterial biofilms: from the natural environment to infectious diseases. Nat Rev Microbiol 2: 95–108. 15040259 10.1038/nrmicro821

[2] Costerton W, Veeh R, Shirtliff M, Pasmore M, Post C, Ehrlich G (2003) The application of biofilm science to the study and control of chronic bacterial infections. J Clin Invest 112: 1466–1477. 14617746 10.1172/JCI20365 PMC259139

[3] Rupnik M, Wilcox MH, Gerding DN (2009) *Clostridium difficile* infection: new developments in epidemiology and pathogenesis. Nat Rev Microbiol 7: 526–536. 10.1038/nrmicro2164 19528959

[4] Mergenhagen KA, Wojciechowski AL, Paladino JA (2014) A review of the economics of treating *Clostridium difficile* infection. Pharmacoeconomics 32: 639–650. 10.1007/s40273-014-0161-y 24807468

[5] Loo VG, Poirier L, Miller MA, Oughton M, Libman MD, Michaud S, et al (2005) A predominantly clonal multi-institutional outbreak of *Clostridium difficile*-associated diarrhea with high morbidity and mortality. N Engl J Med 353: 2442–2449. 16322602 10.1056/NEJMoa051639

[6] Fagan RP, Janoir C, Collignon A, Mastrantonio P, Poxton IR, Fairweather NF (2011) A proposed nomenclature for cell wall proteins of *Clostridium difficile* . J Med Microbiol 60: 1225–1228. 10.1099/jmm.0.028472-0 21252271

[7] Janoir C, Pechine S, Grosdidier C, Collignon A (2007) Cwp84, a surface-associated protein of *Clostridium difficile*, is a cysteine protease with degrading activity on extracellular matrix proteins. J Bacteriol 189: 7174–7180. 17693508 10.1128/JB.00578-07 PMC2168428

[8] ChapetonMontes D, Candela T, Collignon A, Janoir C (2011) Localization of the *Clostridium difficile* cysteine protease Cwp84 and insights into its maturation process. J Bacteriol 193: 5314–5321. 10.1128/JB.00326-11 21784932 PMC3187449

[9] de la Riva L, Willing SE, Tate EW, Fairweather NF (2011) Roles of cysteine proteases Cwp84 and Cwp13 in biogenesis of the cell wall of *Clostridium difficile* . J Bacteriol 193: 3276–3285. 10.1128/JB.00248-11 21531808 PMC3133288

[10] Kirby JM, Ahern H, Roberts AK, Kumar V, Freeman Z, Acharya KR, et al (2009) Cwp84, a surface-associated cysteine protease, plays a role in the maturation of the surface layer of *Clostridium difficile* . J Biol Chem 284: 34666–34673. 10.1074/jbc.M109.051177 19808679 PMC2787329

[11] Dapa T, Leuzzi R, Ng YK, Baban ST, Adamo R, Kuehne SA, et al (2013) Multiple factors modulate biofilm formation by the anaerobic pathogen *Clostridium difficile* . J Bacteriol 195: 545–555. 10.1128/JB.01980-12 23175653 PMC3554014

[12] Dawson LF, Valiente E, Faulds-Pain A, Donahue EH, Wren BW (2012) Characterisation of *Clostridium difficile* biofilm formation, a role for Spo0A. PLoS One 7: e50527 10.1371/journal.pone.0050527 23236376 PMC3517584

[13] Donelli G, Vuotto C, Cardines R, Mastrantonio P (2012) Biofilm-growing intestinal anaerobic bacteria. FEMS Immunol Med Microbiol 65: 318–325. 10.1111/j.1574-695X.2012.00962.x 22444687

[14] Semenyuk EG, Laning ML, Foley J, Johnston PF, Knight KL, Gerding DN, et al (2014) Spore formation and toxin production in *Clostridium difficile* biofilms. PLoS One 9: e87757 10.1371/journal.pone.0087757 24498186 PMC3907560

[15] Pantaleon V, Bouttier S, Soavelomandroso AP, Janoir C, Candela T (2014) Biofilms of *Clostridium* species. Anaerobe. 10.1016/j.anaerobe.2014.09.010 25242197

[16] Federle MJ, Bassler BL (2003) Interspecies communication in bacteria. J Clin Invest 112: 1291–1299. 14597753 10.1172/JCI20195 PMC228483

[17] Merrigan MM, Venugopal A, Roxas JL, Anwar F, Mallozzi MJ, Roxas BA, et al (2013) Surface-layer protein A (SlpA) is a major contributor to host-cell adherence of *Clostridium difficile* . PLoS One 8: e78404 10.1371/journal.pone.0078404 24265687 PMC3827033

[18] Giaouris E, Chapot-Chartier MP, Briandet R (2009) Surface physicochemical analysis of natural Lactococcus lactis strains reveals the existence of hydrophobic and low charged strains with altered adhesive properties. Int J Food Microbiol 131: 2–9. 10.1016/j.ijfoodmicro.2008.09.006 18954916

[19] Spigaglia P, Barketi-Klai A, Collignon A, Mastrantonio P, Barbanti F, Rupnik M, et al (2013) Surface-layer (S-layer) of human and animal *Clostridium difficile* strains and their behaviour in adherence to epithelial cells and intestinal colonization. J Med Microbiol 62: 1386–1393. 10.1099/jmm.0.056556-0 23518658

[20] Hamon MA, Lazazzera BA (2001) The sporulation transcription factor Spo0A is required for biofilm development in *Bacillus subtilis* . Mol Microbiol 42: 1199–1209. 11886552 10.1046/j.1365-2958.2001.02709.x

[21] Crowther GS, Chilton CH, Todhunter SL, Nicholson S, Freeman J, Baines SD, et al (2014) Comparison of planktonic and biofilm-associated communities of *Clostridium difficile* and indigenous gut microbiota in a triple-stage chemostat gut model. J Antimicrob Chemother 69: 2137–2147. 10.1093/jac/dku116 24788662

[22] Miller JH (1972) Experiments in molecular genetics Cold Spring Harbor Laboratory, Cold Spring Harbor, NY.

[23] Hussain HA, Roberts AP, Mullany P (2005 ) Generation of an erythromycin-sensitive derivative of *Clostridium difficile* strain 630 (630Deltaerm) and demonstration that the conjugative transposon Tn916DeltaE enters the genome of this strain at multiple sites. J Med Microbiol 54: 137–141. 15673506 10.1099/jmm.0.45790-0

[24] Stubbs SL, Brazier JS, O'Neill GL, Duerden BI (1999) PCR targeted to the 16S-23S rRNA gene intergenic spacer region of *Clostridium difficile* and construction of a library consisting of 116 different PCR ribotypes. J Clin Microbiol 37: 461–463. 9889244 10.1128/jcm.37.2.461-463.1999 PMC84342

[25] Bryksin AV, Matsumura I (2010) Overlap extension PCR cloning: a simple and reliable way to create recombinant plasmids. Biotechniques 48: 463–465. 10.2144/000113418 20569222 PMC3121328

[26] Bouillaut L, McBride SM, Sorg JA (2011) Genetic manipulation of *Clostridium difficile* . Curr Protoc Microbiol Chapter 9: Unit 9A 2. 10.1002/9780471729259.mc09a02s20 PMC3615975 21400677

[27] McQuade R, Roxas B, Viswanathan VK, Vedantam G (2012) *Clostridium difficile* clinical isolates exhibit variable susceptibility and proteome alterations upon exposure to mammalian cationic antimicrobial peptides. Anaerobe 18: 614–620. 10.1016/j.anaerobe.2012.09.004 23017940

[28] Calabi E, Ward S, Wren B, Paxton T, Panico M, Morris H, et al (2001) Molecular characterization of the surface layer proteins from *Clostridium difficile* . Mol Microbiol 40: 1187–1199. 11401722 10.1046/j.1365-2958.2001.02461.x

[29] Wexler H, Mulligan ME, Finegold SM (1984) Polyacrylamide gel electrophoresis patterns produced by *Clostridium difficile* . Rev Infect Dis 6 Suppl 1: S229–234. 6718936 10.1093/clinids/6.supplement_1.s229

[30] Pawitan Y, Michiels S, Koscielny S, Gusnanto A, Ploner A (2005) False discovery rate, sensitivity and sample size for microarray studies. Bioinformatics 21: 3017–3024. 15840707 10.1093/bioinformatics/bti448

[31] Aziz RK, Bartels D, Best AA, DeJongh M, Disz T, Edwards RA, et al (2008) The RAST Server: rapid annotations using subsystems technology. BMC Genomics 9: 75 10.1186/1471-2164-9-75 18261238 PMC2265698

[32] Fagan RP, Albesa-Jove D, Qazi O, Svergun DI, Brown KA, Fairweather NF (2009) Structural insights into the molecular organization of the S-layer from *Clostridium difficile* . Mol Microbiol 71: 1308–1322. 10.1111/j.1365-2958.2009.06603.x 19183279

[33] Bellon-Fontaine M-N, Rault J, Oss CJv (1996) Microbial adhesion to solvents: a novel method to determine the electron-donor/electron-acceptor or Lewis acid-base properties of microbial cells. Colloids and surfaces B: Biointerfaces 7: 47–53.

[34] Ng YK, Ehsaan M, Philip S, Collery MM, Janoir C, Collignon A, et al (2013) Expanding the repertoire of gene tools for precise manipulation of the *Clostridium difficile* genome: allelic exchange using pyrE alleles. PLoS One 8: e56051 10.1371/journal.pone.0056051 23405251 PMC3566075

[35] Burns DA, Minton NP (2011) Sporulation studies in *Clostridium difficile* . J Microbiol Methods 87: 133–138. 10.1016/j.mimet.2011.07.017 21864584

