# Supplementary material for: The Clostridium difficile Protease Cwp84 Modulates both Biofilm Formation and Cell-Surface Properties
Source: PLoS One. 2015 Apr 29;10(4):e0124971. doi: 10.1371/journal.pone.0124971 (PMC4414356; doi:10.1371/journal.pone.0124971)
Supplement: S1 Fig — Biofilms of R20291, R20291Δcwp84 (CRG2549), R20291Δcwp84+cwp84 (CRG3059) [11], 630Δerm, 630ΔermΔcwp84 (CRG2302) and 630ΔermΔcwp84+cwp84 (CRG2445) [34] strains were quantified using violet crystal. Data are the average of two independent experiments, each performed in technical triplicates. (DOCX) [file pone.0124971.s001.docx]

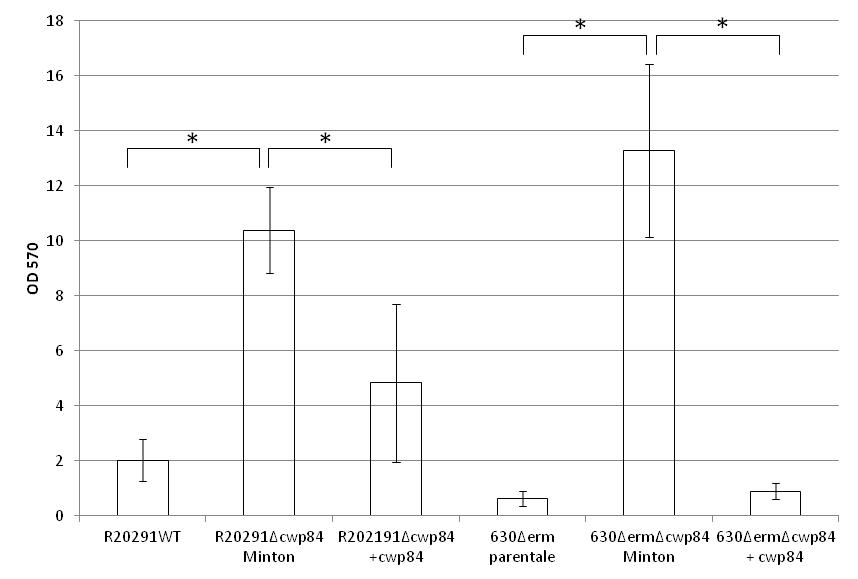


***Figure S1***. **cwp84 *mutants obtained by the allelic exchange method in the 630∆erm and the* R20291 *strains harbor the same phenotype than the* cwp84 *mutant obtained by the clostron method in the 630∆erm strain.***

Biofilms of R20291, R20291*∆cwp84* (CRG2549), R20291*∆cwp84*+*cwp84* (CRG3059) [[1](#_ENREF_1)], 630*∆erm*, 630*∆erm∆cwp84* (CRG2302) and 630*∆erm∆cwp84*+*cwp84* (CRG2445) [[2](#_ENREF_2)] strains were quantified using violet crystal. Data are the average of two independent experiments, each performed in technical triplicates. Significantly different (*p* < 0.01, Student *t* test) ratios are indicated by asterisks.

1. Dapa T, Leuzzi R, Ng YK, Baban ST, Adamo R, et al. (2013) Multiple factors modulate biofilm formation by the anaerobic pathogen Clostridium difficile. J Bacteriol 195: 545-555.

2. Ng YK, Ehsaan M, Philip S, Collery MM, Janoir C, et al. (2013) Expanding the repertoire of gene tools for precise manipulation of the Clostridium difficile genome: allelic exchange using pyrE alleles. PLoS One 8: e56051.
