# Supplementary material for: The Clostridium difficile Protease Cwp84 Modulates both Biofilm Formation and Cell-Surface Properties
Source: PLoS One. 2015 Apr 29;10(4):e0124971. doi: 10.1371/journal.pone.0124971 (PMC4414356; doi:10.1371/journal.pone.0124971)
Supplement: S2 Fig — The 630Δerm versus cwp84 mutant were grown in competition in axenic mice for up to 5 days. In vivo analyses of bacterial (630Δerm in blue, cwp84 mutant in green) and spores (630Δerm in pink, cwp84 mutant in red)counts were performed using feces (A) or via cecal (B) enumeration as described in the Methods. Error bars represent standard deviation. CFUs counts were performed as described in the material and methods, except that the sample was used before and after a 70°C incubation for 30 minutes to differentiate between bacteria and spores as described in Burns and Minton 2011 [35]. (DOCX) [file pone.0124971.s002.docx]

A


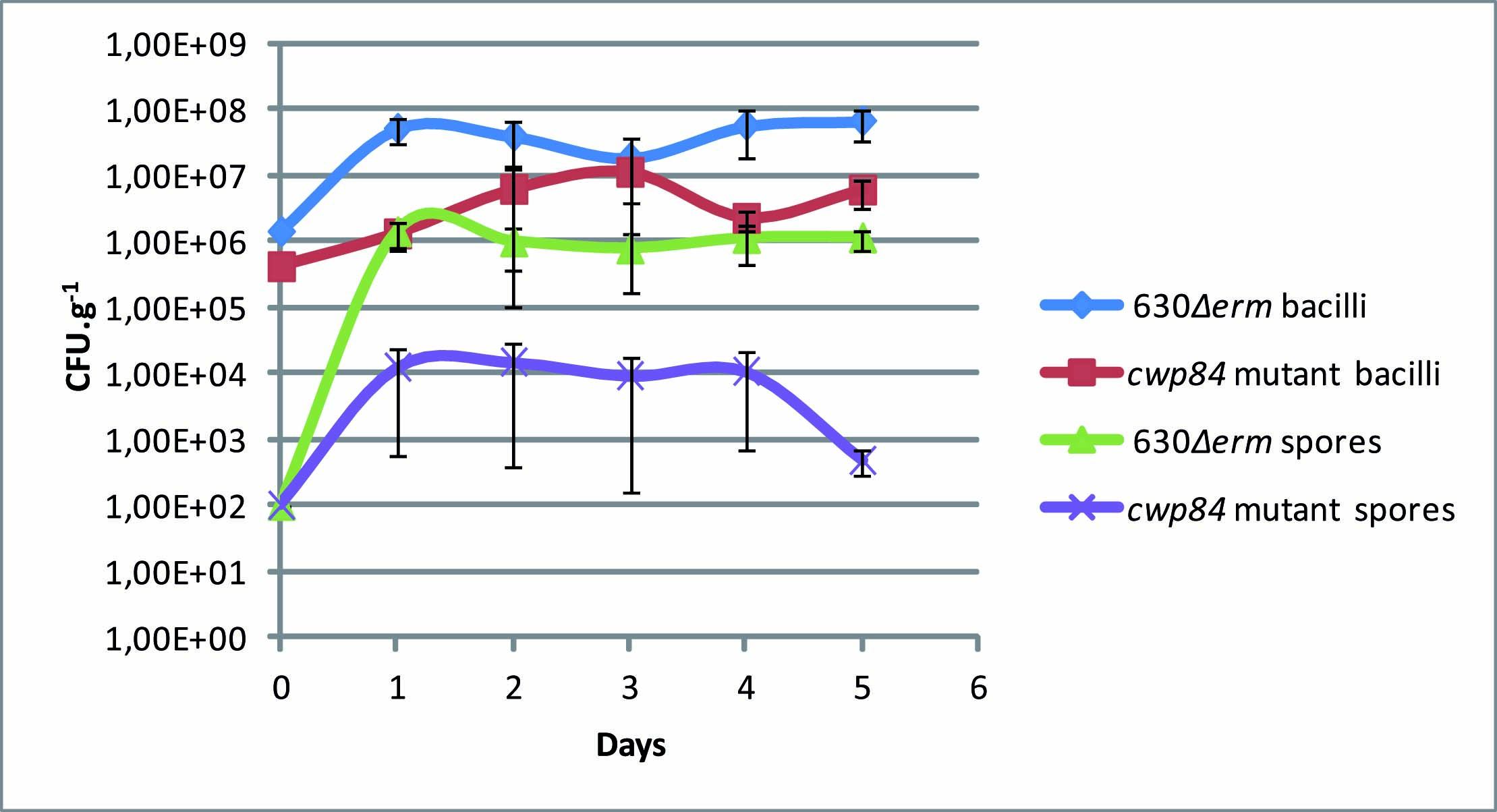


B


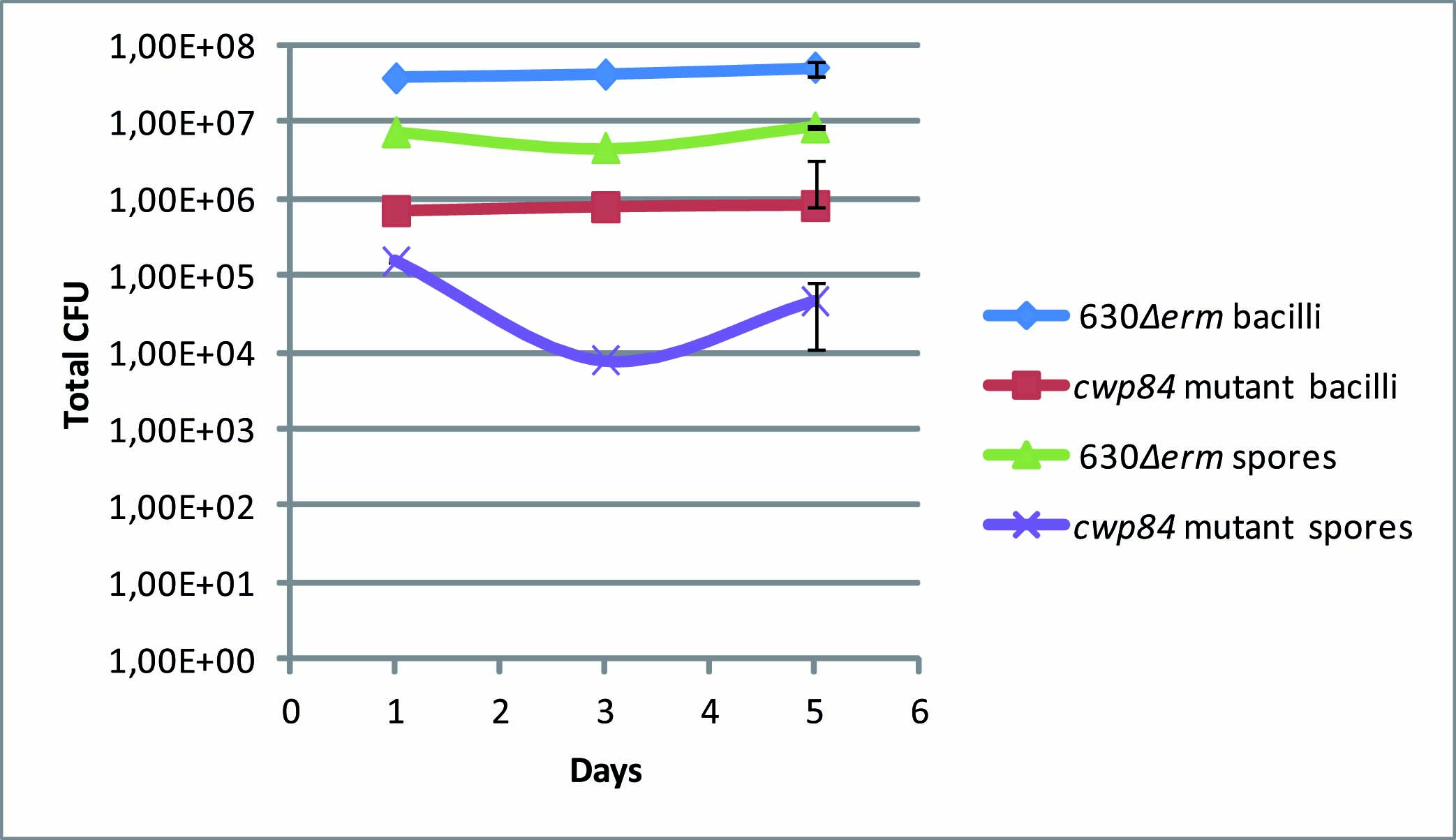


**Figure S2 : In vivo competition of parental and *cwp84* strains, bacilli and spores details**

The 630*∆erm* versus *cwp84* mutant were grown in competition in axenic mice for up to 5 days. In vivo analyses of bacterial (630*∆erm* in blue, *cwp84* mutant in green) and spores (630*∆erm* in pink, *cwp84* mutant in red)counts were performed using feces (A) or via cecal (B) enumeration as described in the Methods. The CFU.g^-1^ at day 0 in the feces (A) represent the bacilli or spore present in the inoculums. Error bars represent standard deviation. CFUs counts were performed as described in the material and methods, except that the sample was used before and after a 70°C incubation for 30 minutes to differentiate between bacteria and spores as described in Burns and Minton 2011 [[1](#_ENREF_1)].

Reference: 1. Burns DA, Minton NP (2011) Sporulation studies in *Clostridium difficile*. J Microbiol Methods 87: 133-138.
