# Supplementary material for: The Clostridium difficile Protease Cwp84 Modulates both Biofilm Formation and Cell-Surface Properties
Source: PLoS One. 2015 Apr 29;10(4):e0124971. doi: 10.1371/journal.pone.0124971 (PMC4414356; doi:10.1371/journal.pone.0124971)
Supplement: S3 Fig — The 630Δerm (blue curves) and cwp84 mutant (red curves) strains were grown separately in agitated planktonic culture. Colony forming units (CFU) were enumerated in three independent experiments and the content of spores and bacilli is presented in the Panel A and B, respectively. Spores and bacilli counts were performed as described in S2 Fig. (DOCX) [file pone.0124971.s003.docx]

A


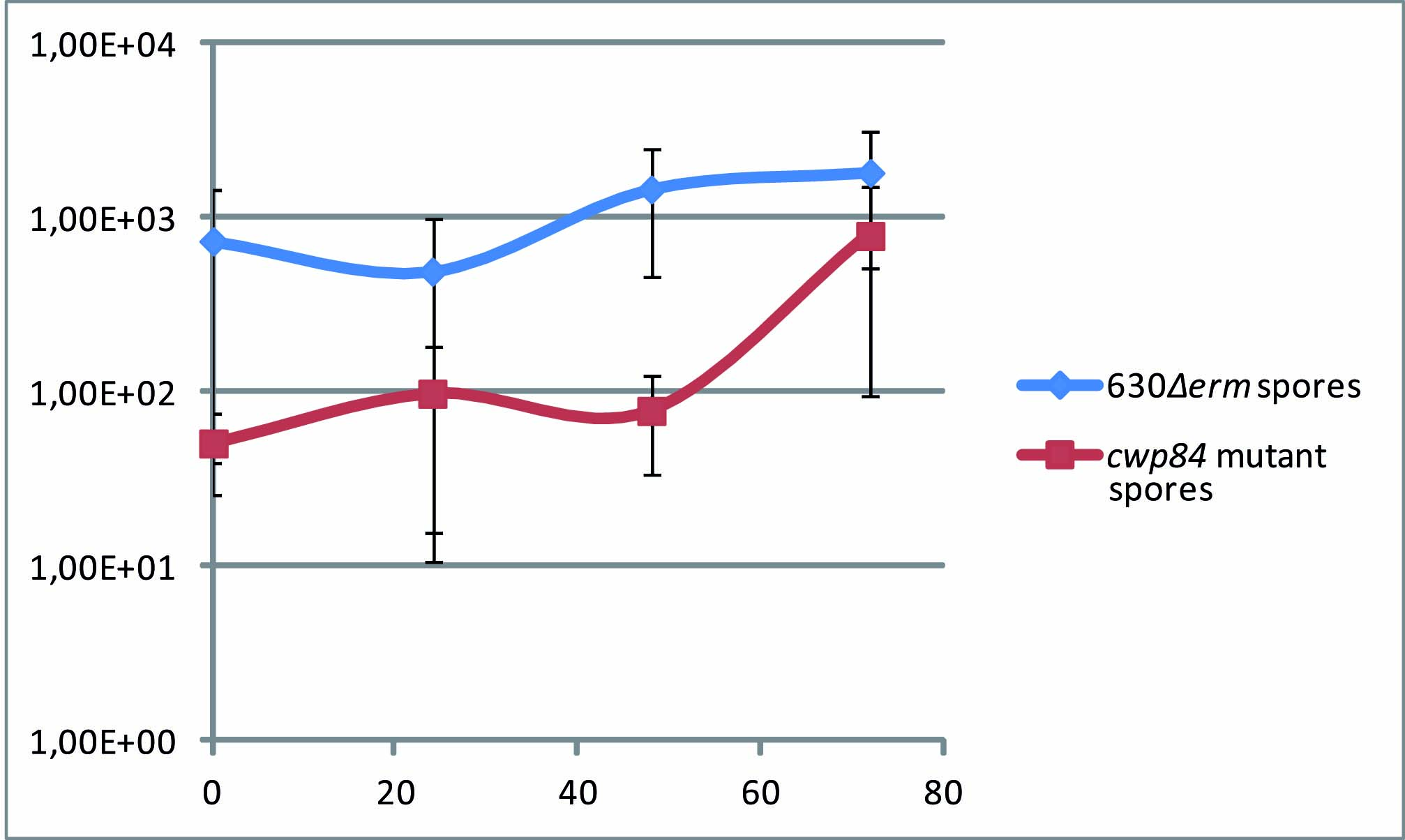


B


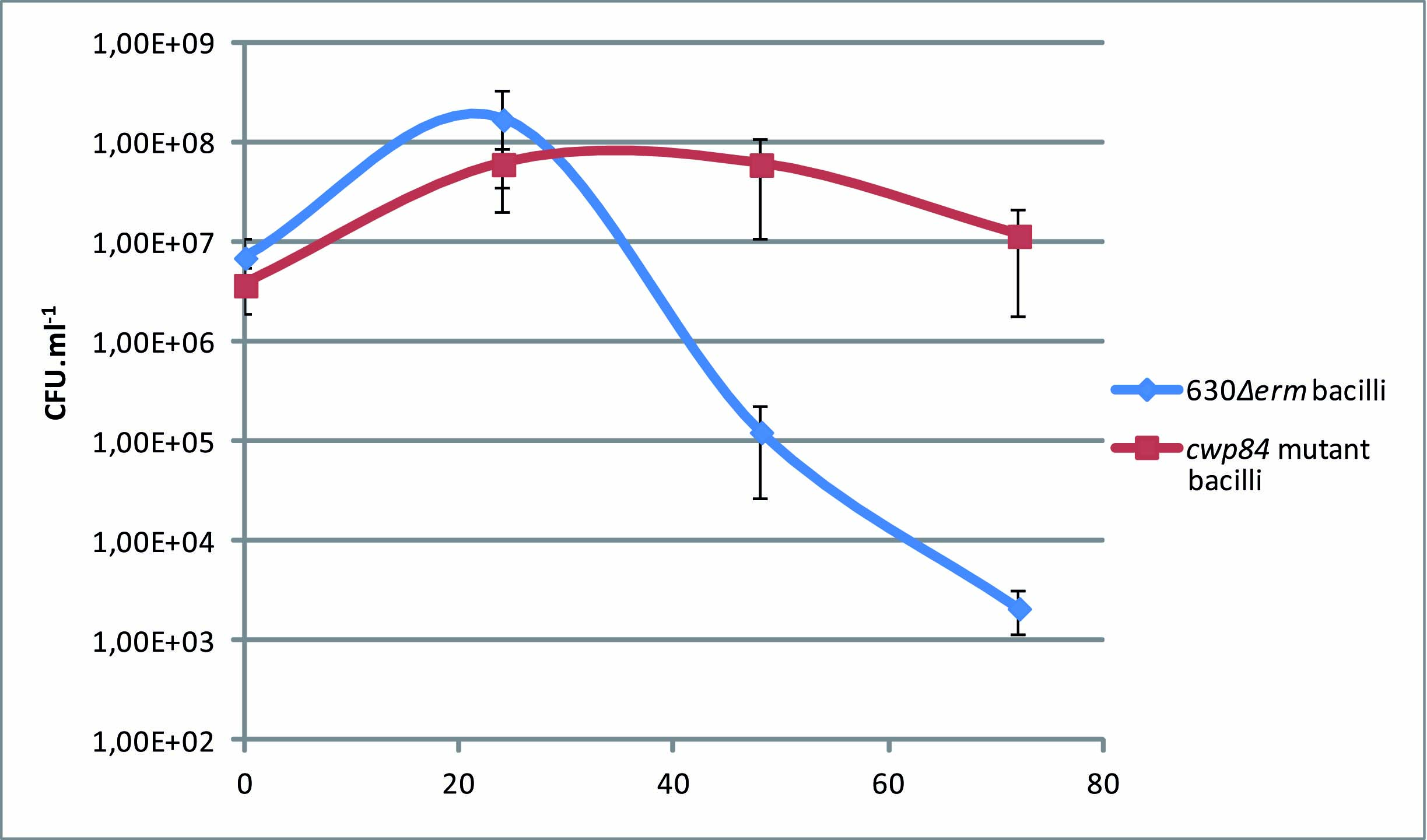


**Figure S3: 630Δ*erm* and *cwp84* mutant kinetics over 72h of growth.**

The 630*∆erm* (blue curves) and *cwp84* mutant (red curves) strains were grown separately in agitated planktonic culture. Colony forming units (CFU) were enumerated in three independent experiments and the content of spores and bacilli is presented in the Panel A and B, respectively. Spores and bacilli counts were performed as described in Figure S2.
